# Preferences for Telehealth Physical Activity Participation Among a Cohort of Children and Youth With Disabling Conditions: Cross-Sectional Survey Study

**DOI:** 10.2196/82854

**Published:** 2026-06-05

**Authors:** Byron Lai, Katie Bonds, Tracy Flemming Tracy, Yumi Kim, Hui-Ju Young, James H Rimmer, Jennifer Angeli

**Affiliations:** 1 Department of Pediatrics, University of Alabama at Birmingham, Division of Pediatric Rehabilitation Medicine, 1720 2nd Ave South, Birmingham, AL, 35294, United States, 1 (205) 934-4011; 2 Division of Occupational Therapy and Physical Therapy, Cincinnati Children’s Hospital Medical Center, Cincinnati, OH, United States; 3 Department of Physical and Occupational Therapy, University of Alabama at Birmingham, Birmingham, AL, United States; 4 Department of Physical Therapy, University of Alabama at Birmingham, Birmingham, AL, United States; 5 Dean's Office, University of Alabama at Birmingham, Birmingham, AL, United States

**Keywords:** telehealth, pediatric rehabilitation, physical activity, tele-exercise, exercise

## Abstract

**Background:**

Children with disabling conditions encounter numerous challenges in participating in physical activity within their community. Telehealth has emerged as an ideal method for promoting physical health and wellness, but there is a need to identify optimal implementation strategies.

**Objective:**

This study aimed to describe the telehealth physical activity preferences of active children and youth with disabling conditions to rapidly inform the development of a pilot telehealth program that could be delivered nationally.

**Methods:**

A cross-sectional survey was conducted among a convenience sample of pediatric members of a community-based wellness program. Questions probed preferences for delivery method; programming frequency, intensity, duration, and type; desired outcomes; technology access and proficiency; and additional needed supports. Of the initial 56 respondents, 4 (7.1%) over the age of 18 years were excluded, leaving 52 (92.9%) for analysis. Outcomes were summarized descriptively.

**Results:**

Of 392 wellness program members, 56 (14.3%) responses were gathered. The mean age of the 52 analyzed respondents was 10 (SD 3; range 5-16) years. The sample predominantly comprised male (32/52, 61.5%) and White (34/52, 65.4%) individuals, with autism spectrum disorder and developmental disorder as the most common disability types (22/52, 42.3% each). Social and psychological barriers were the most frequently reported challenges to physical activity participation (36/52, 69.2% and 27/52, 51.9%, respectively). Most respondents reported an ideal exercise dose of 1 to 2 sessions per week of 30 to 45 minutes at a novice or beginner difficulty level. Winter was the preferred season for participation. The 2 most desired delivery formats were live videoconferencing and prerecorded videos. Desired program outcomes included strength improvement, mental health, developing new hobbies and activities, and social connection. Over 90% of respondents (47/52, 90.4%) reported having adequate technology at home to support virtual participation.

**Conclusions:**

Optimal telehealth programs for this cohort should be brief and low intensity and offered seasonally, with both live and prerecorded delivery options. Although this preferred dose does not meet US physical activity guidelines, it may represent an appropriate starting point for many inactive children with disabilities. Future research should examine behavior change strategies that motivate children to enroll in these programs and support gradual increases in physical activity over time.

## Introduction

Children with disabilities face a high risk of health decline as they age into middle adulthood, largely due to the emergence of secondary health conditions such as obesity and cardiovascular disease [1-4]. This is believed to be caused by persistently low rates of participation in physical activity [5]. Opportunities for physical activity for children with disabilities are largely provided at on-site facilities, parks, and recreation centers. On-site participation, while beneficial, has been found to have numerous barriers (eg, transportation, cost, and accommodation of special needs) that make regular participation difficult for children with disabilities [6].

Digital telehealth programs have emerged as a viable option for promoting physical activity among children with disabilities who cannot attend on-site programs [7-9]. Programs delivered at a facility require transportation, nearby accessible and usable facilities and equipment, and knowledgeable staff [6]. In contrast, telehealth programs can be delivered directly to the home, removing many of the structural barriers that limit access for this population [10,11]. Home-based delivery may also reduce exposure to social and sensory stressors that are commonly reported as barriers among children with disabilities, which may further support sustained engagement over time [12,13].

A number of telehealth physical activity programs have been developed and tested for children with disabilities, with growing evidence supporting their feasibility and acceptability [8,10,14,15]. These programs have used a range of delivery formats, including live videoconferencing with a coach or group, prerecorded exercise videos for independent use, and hybrid models combining both. For example, among children with cerebral palsy, telehealth exercise programs targeting aerobic fitness and physical function have shown early promise [8,15,16], including movement-to-music programs delivered remotely [17,18] and virtual reality tele-exergaming protocols designed to support cardiometabolic health in the home environment [19]. Caregiver and clinician acceptability of these approaches has generally been favorable [10,20].

Little is known about the preferences of children with disabilities for telehealth physical activity program delivery as this remains an emerging area of research [16]. Given the diversity of this population, a range of delivery strategies will likely be needed to accommodate varying needs and preferences [21]. Therefore, understanding what children with disabilities prefer in a telehealth context is an important step toward developing programs with stronger uptake and engagement.

The purpose of this study was to rapidly describe the telehealth physical activity programming preferences of a cohort of children with disabilities to inform remotely delivered intervention designs.

## Methods

### Design and Setting

This study was a time-limited, cross-sectional survey of children who were reached through a pediatric rehabilitation team from a single midwestern children’s hospital in the United States. All survey respondents had previously participated in be.well, a community-based wellness program that provides locally adapted physical activity opportunities for children with chronic or complex medical conditions. The be.well program offers approximately two dozen programs that include 40 community partners and 80 volunteers, delivering programming during more than 75% of the weeks throughout a calendar year. Recruitment took place over 2 quarters (September 2024-February 2025) using a convenience sampling approach; no minimum survey response rate was set a priori.

The rapid, time-limited design was selected to generate timely, actionable insights to support the codevelopment of a pilot community program in partnership between the hospital and the National Center on Health, Physical Activity, and Disability (NCHPAD). The NCHPAD provides a large variety of no-cost, online health promotion programs to thousands of adults across the United States. In partnership with be.well, the NCHPAD aimed to obtain information to guide the development of a national, child-focused model for implementing web-based physical activity programs. Rapid assessment approaches are recognized as appropriate when the research goal is descriptive and actionable rather than confirmatory, particularly in the early stages of program development [22].

### Participants

Study invitations were distributed via the be.well listserve. Emails included a public survey link that automatically captured responses in a secure web platform, REDCap (Research Electronic Data Capture; Vanderbilt University). The eligibility criteria were as follows: (1) being younger than 18 years; (2) having a disabling, chronic, or complex condition (eg, physical, cognitive, or visual disability or developmental delay); and (3) having previously participated in at least one be.well program.

### Ethical Considerations

This study was determined to be an opt-out protocol by the institutional review board of the University of Alabama at Birmingham (IRB-300008580). Written informed consent and assent were deemed not necessary for participation in the study if prospective participants were informed about the study and given a clear opportunity to decline participation. Study data were deidentified using alphanumeric codes, and participation was confidential. No compensation was provided for participation.

### Survey Design

The survey was developed collaboratively by the NCHPAD and be.well teams, which included 5 clinical rehabilitation specialists with expertise in adapted physical activity, pediatric rehabilitation, and telehealth program delivery. Survey items were generated through a structured consensus process in which team members independently drafted candidate items based on their clinical experience and a review of existing literature on telehealth preferences and barriers in pediatric populations. Items were subsequently reviewed and refined through group discussion until consensus was reached on final item wording and response options. The final survey was administered in English only.

Respondents were recommended to complete the survey jointly (family member and child), although responses submitted by either the child or their family member alone were eligible for inclusion. The survey included 2 sections: participant information and preferences for telehealth exercise participation. Participant information captured demographics (age, sex, race, and ethnicity) and functional characteristics (disability type; mobility limitation; assistive device use; and barriers to exercise participation, physical function, communication style, vision function, and hearing function). The preference section probed the following areas: delivery method (in person or virtual); programming frequency, intensity, duration, and type; desired outcomes; technology proficiency and resources; and additional needed supports. Most items used forced-choice or multiple-selection response formats; 1 open-ended item was included to capture additional comments.

### Analysis

Participant demographics and characteristics, as well as all survey responses, were summarized using descriptive statistics. Continuous variables are reported as means and SDs; categorical variables are reported as frequencies and percentages. For multiple-selection items, proportions were calculated relative to the total number of respondents who answered that item. Subgroup descriptive comparisons were made between respondents who expressed interest in virtual programming and those who did not to characterize any apparent differences in preferences by delivery format interest. No inferential statistical comparisons were established a priori, consistent with the descriptive intent of the study. Descriptive data were managed and analyzed using REDCap’s native export functionality and Microsoft Excel.

## Results

### Respondents and Characteristics

From emails to 392 be.well participants, 56 (14.3%) responses were obtained. Of these 56 respondents, 4 (7.1%) were excluded due to age. The characteristics of the 52 respondents analyzed are shown in Table 1. Most surveys were completed by a caregiver or family member (22/52, 42.3% and 27/52, 51.9%, respectively), with 5.8% (3/52) completed by the children themselves. The mean age of the respondents was 10 (SD 3) years, with ages ranging from 5 to 16 years. Respondents were predominantly male (32/52, 61.5%) and White individuals (34/52, 65.4%), with 42.3% (22/52) reporting autism spectrum disorder and 42.3% (22/52) reporting a developmental disorder. Most (51/52, 98.1%) lived in urban environments. A total of 36.5% (19/52) of respondents had a mobility limitation, and just over half (10/19, 52.6%) of them used at least one assistive device. Social (36/52, 69.2%) and psychological barriers (27/52, 51.9%) were identified as the most frequent challenges to physical activity participation. Access (17/52, 32.7%), physical (16/52, 30.8%), financial (15/52, 28.8%), and institutional barriers (15/52, 28.8%) were commonly reported, whereas informational barriers were the least notable at 21.2% (11/52). Specific barriers are described in Table 1.

**Table 1.** Respondents’ characteristics (N=52).

Category	Values
Survey respondent, n (%)	
Caregiver	22 (42.3)
Family member	27 (51.9)
Child	3 (5.8)
Age (y), mean (SD; range)	10 (3; 5-16)
Sex, n (%)	
Male	32 (61.5)
Female	20 (38.5)
Ethnicity (Hispanic, Latino, or of Spanish origin), n (%)	
Yes	2 (3.8)
No	48 (92.3)
Prefer not to answer	2 (3.8)
Race, n (%)^a^	
Asian	4 (7.7)
Black or African American	5 (9.6)
Multiracial	6 (11.5)
White	34 (65.4)
Prefer not to answer	3 (5.8)
Living area, n (%)^b^	
Urban	51 (98.1)
Suburban or large town	0 (0)
Small town	0 (0)
Isolated rural	1 (1.9)
Disability type , n (%)^a^	
Autism spectrum disorder	22 (42.3)
Developmental disorder	22 (42.3)
Cerebral palsy	14 (26.9)
Speech disorder	5 (9.6)
Sensory processing disorder	3 (5.8)
Having a twin sibling with a disability, n (%)	
Yes	1 (1.9)
No	51 (98.1)
Presence of mobility limitations, n (%)	
Yes	19 (36.5)
No	33 (63.5)
Use of assistive devices (among those with mobility limitations; n=19), n (%)	
Yes	10 (52.6)
No	9 (47.4)
Type of assistive device (among those with assistive device use; n=10), n (%)^a^	
Orthotics (AFOs^c^)	6 (60)
Manual wheelchair—propelled by the participant	4 (40)
Manual wheelchair—participant is transported	3 (30)
Walker	2 (20)
Gait trainer	1 (10)
Manual ability, n (%)	
Handles objects easily and successfully	20 (38.5)
Handles most objects but with reduced quality	26 (50)
Handles objects with difficulty; needs help	5 (9.6)
Handles limited objects in adapted situations	1 (1.9)
Does not handle objects	0 (0)
Speech communication ability, n (%)	
Effective sender or receiver with everyone	23 (44.2)
Effective but slower-paced sender or receiver	12 (23.1)
Effective sender or receiver with known individuals	7 (13.5)
Inconsistent sender or receiver with known individuals	7 (13.5)
Seldom effective sender or receiver	3 (5.8)
Vision function, n (%)	
Uses visual function easily and successfully	41 (78.8)
Uses visual function successfully with help	1 (1.9)
Uses visual function but needs adaptations	5 (9.6)
Uses visual function partly with adaptations	3 (5.8)
Does not use visual function	2 (3.8)
Hearing function, n (%)	
Uses hearing easily and successfully	43 (82.7)
Uses hearing successfully with help	3 (5.8)
Uses hearing but needs adaptations	1 (1.9)
Uses hearing function partly with adaptations	0 (0)
Does not use hearing function	5 (9.6)
Perceived barriers to exercise participation, n (%)^a^	
Social (inclusive programming; social stigma or attitudes; exclusion by others; and lack of understanding of coaches, teachers, and staff)	36 (69.2)
Psychological (low self-esteem, anxiety regarding socialization, and fear of failure)	27 (51.9)
Sensory (visual or hearing-related challenges)	20 (38.5)
Access (transportation, facility access, and nearby specialists and programs)	17 (32.7)
Physical (limited mobility; need for specialized equipment; and pain, fatigue, or other health-related issues)	16 (30.8)
Financial (high cost of adaptive equipment or additional support)	15 (28.8)
Institutional (lack of trained staff, inadequate policies for inclusion, and limited funding for inclusive recreation)	15 (28.8)
Informational (lack of knowledge of programs and inadequate communication between caregiver and service providers)	11 (21.2)
Other^d^	4 (7.7)

a Respondents could select more than one response option.

b Residential location was classified using the Rural-Urban Commuting Area codes based on participants’ zip codes, reflecting population density, urbanization, and commuting patterns.

c AFO: ankle-foot orthosis.

d Other reasons for perceived barriers to exercise: time (caregiver work schedules), distraction, almost no one wearing masks or making places safe to share space with others, and looking for inclusive teenager programs.

### Preference Summary

Table 2 presents telehealth programming preferences and barriers to virtual participation. Of the 52 respondents who answered this item, 32 (61.5%) reported interest in participating in virtual programming. The remaining 38.5% (20/52) preferred in-person sessions and indicated that they would likely lose interest if the program were delivered virtually. Among those favoring the in-person format, 20% (4/20) noted a need for hands-on support from volunteers or staff for suitable participation.

**Table 2.** Teleheath programming preference and barriers to participation (N=52).

	Participants interested in virtual programming, n (%)		
	Yes (n=32)	No (n=20)	Total
Reasons for not being interested^a^			
Preferred in-person programming	—^b^	18 (90)	—
Lost interest in video or virtual programming	—	12 (60)	—
Need for hands-on support from volunteers and staff	—	4 (20)	—
Did not have the proper home setup (eg, internet or computer)	—	0 (0)	—
Lack of technological knowledge or familiarity	—	0 (0)	—
Current technology setup and necessary skills			
Sufficient in both cases	30 (93.8)	17 (85)	47 (90.4)
Sufficient technology setup but limited skill	0 (0)	1 (5)	1 (1.9)
Limited technology setup but sufficient skill	1 (3.1)	1 (5)	2 (3.8)
Limited in both cases	1 (3.1)	1 (5)	2 (3.8)

a Respondents could select more than one response option.

b Not applicable.

A total of 90.4% (47/52) of the respondents reported having sufficient supplies at home and skill to support telehealth communications. In total, 1.9% (1/52) of the respondents reported sufficient technology but limited operational skill, 3.8% (2/52) reported limited technology setup but sufficient skill, and 3.8% (2/52) reported insufficient supplies and insufficient operational skill.

### Desired Program Characteristics

The desired virtual programming characteristics for the 32 interested respondents are shown in Table 3. A total of 81.3% (n=26) of the respondents expressed a desire for an exercise and fitness class. Most respondents were interested in participating in a virtual martial arts (n=25, 78.1%), yoga (n=23, 71.9%), or dance (n=19, 59.4%) program. Respondents most commonly desired virtual programming in the winter (n=32, 100%), followed by summer (n=22, 68.8%). On the basis of survey responses, an ideal program is one that improves physical strength (n=25, 78.1%), provides new experiences and hobbies (n=22, 68.8%), improves how a person feels and thinks (n=20, 62.5%), and provides a sense of belonging or friendship (n=19, 59.4%). Notable but less commonly reported desired benefits included improvement in energy (n=12, 37.5%) and management of health conditions (n=12, 37.5%). Sleep quality was also identified as a notable concern, with 28.1% (n=9) of the virtual programming–interested sample reporting this as a desired outcome.

**Table 3.** Desired programming (N=52).

	Participants interested in virtual programming, n (%)		
	Yes (n=32)	No (n=20)	Total
Type of preferred virtual programming^a^			
Exercise and fitness	26 (81.3)	—^b^	—
Martial arts	25 (78.1)	—	—
Yoga	23 (71.9)	—	—
Dance	19 (59.4)	—	—
Preferred season for virtual programming^a^			
Winter	32 (100)	16 (80)	48 (92.3)
Summer	22 (68.8)	6 (30)	28 (53.8)
Spring	14 (43.8)	3 (15)	17 (32.7)
Fall	10 (31.3)	4 (20)	14 (26.9)
What would you like to get from the program?^a^			
I want to improve my strength.	25 (78.1)	8 (40)	33 (63.5)
I want to find new hobbies or fun things to do.	22 (68.8)	13 (65)	35 (67.3)
I want to improve how I feel and think (eg, less depressed, anxious, and nervous, or feel better about yourself).	20 (62.5)	8 (40)	28 (53.8)
I am looking for a sense of belonging/friendship.	19 (59.4)	14 (70)	33 (63.5)
I want to improve how much energy I have.	12 (37.5)	3 (15)	15 (28.8)
I want to help a health condition (eg, spasticity, diabetes, blood pressure, asthma, pain).	12 (37.5)	8 (40)	20 (38.5)
I want to improve how I sleep.	9 (28.1)	5 (25)	14 (26.9)
I want to improve my ability to walk.	2 (6.3)	0 (0)	2 (3.8)
I want to do everyday tasks better.	0 (0)	0 (0)	0 (0)
Preferred virtual programming delivery format^a^			
Videoconferencing group classes with one or more people over the internet (eg, Zoom or Skype)	26 (81.3)	10 (50)	36 (69.2)
Prerecorded videos to do independently without another person	19 (59.4)	6 (30)	25 (48.1)
SMS text message	3 (9.4)	0 (0)	3 (5.8)
Mobile phone call	3 (9.4)	4 (20)	7 (13.5)
None of the above (virtual option did not work for them)	0 (0)	5 (25)	5 (9.6)
Session frequency per week			
2	14 (43.8)	0 (0)	14 (26.9)
1	13 (40.6)	18 (90)	31 (59.6)
3	3 (9.4)	2 (10)	5 (9.6)
≥5	2 (6.3)	0 (0)	2 (3.8)
Session duration (min)			
30	17 (53.1)	8 (40)	25 (48.1)
45	12 (37.5)	1 (5)	13 (25)
60	2 (6.3)	6 (30)	8 (15.4)
15	1 (3.1)	4 (20)	5 (9.6)
90	0 (0.0)	1 (5)	1 (1.9)
Difficulty level			
Novice or beginner	25 (78.1)	15 (75)	40 (76.9)
Intermediate	7 (21.9)	5 (25)	12 (23.1)
Advanced	0 (0)	0 (0)	0 (0)

a Respondents could select more than one response option.

b Not applicable.

Respondents who were interested in virtual exercise reported an ideal dose of 30 to 45 minutes of exercise 1 to 2 times per week. Respondents who were not interested largely preferred roughly 15 to 60 minutes of exercise 1 day per week. Both groups preferred novice- or beginner-level classes. The 2 most desired delivery modalities were supervised videoconference with one or more people (26/32, 81.3% of those interested in virtual exercise) and prerecorded videos for independent use (19/32, 59.4%).

## Discussion

### Principal Findings

This study described telehealth physical activity preferences among a cohort of children with disabilities who were active members of a community-based wellness program. Most respondents (32/52, 61.5%) expressed interest in virtual programming, with a preference for brief, low-intensity sessions delivered 1 to 2 times per week. Live videoconferencing and prerecorded videos were the most desired delivery formats. Respondents prioritized strength improvement, new experiences, mental health, and social connection as desired program outcomes. These findings offer a preliminary profile of telehealth physical activity preferences that can inform the design of future programming for this population.

### Interpretation and Comparison to Existing Literature

Just over 60% of respondents (32/52, 61.5%) expressed interest in virtual programming, whereas 38.5% (20/52) preferred in-person delivery and indicated that they would disengage if programming moved online. This split is notable and consistent with broader evidence that children with disabilities have highly variable preferences for exercise delivery, driven in part by the diversity of their functional profiles, disability types, and support needs [15,21]. The finding that a meaningful proportion of this sample would not engage virtually reinforces that telehealth should complement rather than replace on-site programming.

The preferred exercise dose (1 to 2 sessions per week of 30 to 45 minutes at a novice or beginner difficulty level) was modest relative to national physical activity guidelines, which recommend a minimum of 150 minutes of moderate-intensity activity per week [23]. This preference gap is not unexpected given the social, psychological, and sensory barriers that children with disabilities commonly report as challenges to participation [6,24]. It is important to note that the preferred dose would likely fall short of thresholds associated with certain health outcomes (eg, cardiometabolic health). However, for children with disabilities, consistent engagement with any structured physical activity may represent a meaningful improvement over baseline as people with disabilities have been shown to benefit from exercise at levels below national guidelines [24]. If higher virtual exercise doses are needed to improve health, program goals should likely focus on engagement, enjoyment, and habit formation for building volume over time. Alternatively, virtual programming could also serve as a supplement to on-site participation for those seeking higher exercise volumes.

Winter was the most strongly preferred period for virtual participation, which likely coincides with school holiday schedules and reduced competing demands on families. Summer was the second most preferred season. This seasonal pattern has practical implications for program scheduling and suggests that short-burst, seasonal telehealth offerings may be more feasible and better attended than year-round programs for this cohort. This is an area that has received little attention in the existing literature on telehealth for children with disabilities and warrants further exploration. One promising direction involves structuring virtual programming around seasonal rotations that mirror the rhythm of community-based sports and recreation programs. For example, fall sessions could feature soccer-based movement activities; winter sessions could emphasize basketball-style games; and spring or summer sessions could incorporate baseball, softball, or track and field activities. Alternatively, programming could be organized around activity type rather than sport, with yoga or low-intensity movement offered in the winter months and more dynamic activities such as martial arts introduced in the spring. The core principle in either approach is variety aligned with the seasonal expectations that families already have of community sport participation.

The 2 most preferred delivery formats were live videoconferencing (26/32, 81.3%) and prerecorded videos (19/32, 59.4%), with mobile-based options endorsed by fewer than 10% of respondents (3/32, 9.4%). These preferences map well onto modalities already represented in the existing literature on children with disabilities [8,10,14,15]. The strong preference for live videoconferencing is notable given that social and psychological barriers were the most commonly reported challenges to participation in this sample (36/52, 69.2% and 27/52, 51.9%, respectively) and that social belonging and mental health improvement were among the top desired outcomes (19/32, 59.4% and 20/32, 62.5% of virtual programming–interested respondents, respectively). This suggests that, for this cohort, the appeal of live formats may extend beyond exercise delivery itself as the structured social contact may be as motivating as the physical activity. Prerecorded videos were endorsed by just under 60% of virtual programming–interested respondents (19/32, 59.4%), likely reflecting the practical reality that families managing complex care schedules need flexible, on-demand options that do not require real-time coordination [12]. The relatively low endorsement of phone-based formats suggests that participants were comfortable with internet-based platforms, which is consistent with the finding that over 90% (47/52, 90.4%) reported having adequate technology at home. Taken together, these preferences suggest that a hybrid model combining live and prerecorded components would serve the broadest range of participants and accommodate both the social and logistical drivers of engagement.

Strength improvement, mental health, new hobbies, and social belonging were the most commonly identified desired outcomes. These outcomes extend beyond what is commonly targeted in telehealth intervention research for children with disabilities [8,10,14,15] and suggests that families in this cohort sought programming that addressed a more holistic approach. The high endorsement of social belonging as a desired outcome is particularly worth noting given that social and psychological barriers were the most frequently reported challenges to participation in this sample. Telehealth programs that intentionally incorporate peer interaction, such as group videoconference formats, may be better positioned to address these motivational drivers and support sustained engagement [25].

For active children in the be.well program, the ideal virtual exercise program would likely be offered in the winter and summer months, which often coincide with school holiday periods. The preferred dose of virtual programming (session frequency, intensity, and duration) appeared to be modest, consisting of 1 to 2 sessions of 30 to 45 minutes per week. Typical telerehabilitation interventions often exceed 60 minutes per week [11]. While this level of exercise may not meet the national guidelines of 150 minutes of moderate-intensity physical activity per week [26], it may represent an appropriate starting point for inactive children with disabilities. Over time, exercise volume could be progressively increased using behavioral economics strategies designed to motivate and encourage higher levels of physical activity. Nevertheless, research suggests that people with disabilities can experience meaningful health benefits from exercise even at levels below national physical activity guidelines [24].

### Limitations

This study had limitations. First, given the small sample size and narrow context (active and engaged members of a community physical activity program), the study findings have limited generalizability. Second, most surveys were completed by caregivers or family members rather than the children themselves, which may not fully reflect the preferences and perspectives of the children. Third, the exercise modalities offered in the survey were limited to those preselected by the study team based on existing be.well programming, restricting the range of options that respondents could express preferences for. Fourth, nearly all respondents (51/52, 98.1%) were from an urban midwestern setting, which may have influenced preferences such as seasonal timing in ways that would not generalize to other regions. Fifth, the 14.3% (56/392) response rate, while acceptable for a rapid survey design, introduces the possibility of response bias, with engaged and motivated families more likely to have completed the survey.

### Conclusions

To our knowledge, this is the first study to examine the telehealth physical activity preferences of children with disabilities who were already engaged in community-based exercise. For this cohort, brief, low-intensity, and seasonally delivered programs that incorporate both live and prerecorded formats appear to be the most acceptable approach. Effective telehealth programming for this population will likely need to go beyond exercise dose and format to address the social and psychological dimensions of participation that this sample clearly valued. Programs developed without input from children and families risk missing motivational drivers. Future research should prioritize reaching children with disabilities who are not currently engaged in any physical activity programming, increase the number of respondents, and identify behavioral economics strategies that increase motivation and engagement in this population.

## Abbreviations

NCHPAD: National Center on Health, Physical Activity, and Disability
REDCap: Research Electronic Data Capture
